# Effect of different tube feeding methods on gastroesophageal reflux features in preterm infants: a pH-impedance monitoring study

**DOI:** 10.1007/s00431-024-05737-7

**Published:** 2024-08-30

**Authors:** Silvia Martini, Fabio Meneghin, Arianna Aceti, Nadia Cerchierini, Isadora Beghetti, Gianluca Lista, Luigi Corvaglia

**Affiliations:** 1 Department of Medical and Surgical Sciences, University of Bologna, Bologna, Italy; 2 Neonatal Intensive Care Unit, IRCCS AOUBO, Bologna, Italy; 3 Neonatology and Neonatal Intensive Care Unit, “V. Buzzi” Children’s Hospital, ASST FBF-Sacco-Buzzi, Milan, Italy

**Keywords:** Continuous tube feeding, Bolus tube feeding, Gastroesophageal reflux, Preterm infants, PH and multiple intraluminal impedance monitoring

## Abstract

A stepwise approach is currently considered the best choice to manage gastroesophageal reflux (GER) in preterm infants. This study aimed to evaluate the effect of different tube feeding techniques on GER frequency and features in symptomatic tube-fed preterm neonates. Tube-fed infants < 34 weeks’ gestation were eligible for this prospective, bicentric, cross-over study if, due to GER symptoms, they underwent a diagnostic 24-h combined pH and multiple intraluminal impedance (pH-MII) monitoring. During the monitoring period, each infant received the same feeding cycle, repeated twice: continuous tube feeding, bolus feeding followed by tube feeding permanence and by tube feeding removal. The impact of these three feeding modalities on pH-MII GER features was assessed. Thirty-one infants were enrolled. Despite a low number of reflux episodes, a significant decrease in total GERs (*P* < 0.001), in GERs detected by pH monitoring (*P* < 0.001), and in both acid and non-acid GERs detected by MII (*P* < 0.001 and *P* = 0.009, respectively) was observed in association with continuous feeding compared to bolus feeds, followed or not by tube feeding removal. Compared to continuous feeding, both bolus feeding modalities were associated with a significantly higher number of proximal GERs (*P* < 0.001). No difference in any pH-MII parameter was observed in relation to tube feeding persistence after bolus feeding administration.

*Conclusions*: Continuous feeding and boluses may have a different impact on pH-MII GER features in symptomatic tube-fed preterm infants, whereas the permanence of the feeding tube across LES did not seem to worsen GER indexes.

**Table:** 

**What is Known**:
• *Due to the functional and anatomical immaturity of the gastrointestinal tract, gastroesophageal reflux (GER) is common in preterm infants.* • *A stepwise therapeutical approach which firstly undertakes conservative strategies is the most advisable choice to avoid potentially harmful pharmacological overtreatments in the preterm population.*
**What is New**:
• *Continuous feeding and boluses may have a different impact on GER features assessed by pH-MII monitoring in tube-fed preterm infants.* • *The permanence of the feeding tube during or after the feeding period did not seem to worsen GER occurrence.* • *By reducing GER features, especially acid GER, continuous feeding may potentially contribute to limit the need for antiacid medications in this population.*

• *Due to the functional and anatomical immaturity of the gastrointestinal tract, gastroesophageal reflux (GER) is common in preterm infants.*

• *A stepwise therapeutical approach which firstly undertakes conservative strategies is the most advisable choice to avoid potentially harmful pharmacological overtreatments in the preterm population.*

• *Continuous feeding and boluses may have a different impact on GER features assessed by pH-MII monitoring in tube-fed preterm infants.*

• *The permanence of the feeding tube during or after the feeding period did not seem to worsen GER occurrence.*

• *By reducing GER features, especially acid GER, continuous feeding may potentially contribute to limit the need for antiacid medications in this population.*

## Introduction

Due to the functional and anatomical immaturity of the gastrointestinal tract, gastroesophageal reflux (GER) is very common in preterm infants. Combined pH and multiple intraluminal impedance monitoring (pH-MII) is currently considered the gold-standard technique to diagnose GER in the pediatric population, as it allows to identify both acid and non-acid GER and to evaluate the esophageal height reached by each GER episode [1].

In symptomatic preterm infants, however, such medications as histamine2-receptor antagonists and proton pump inhibitors are increasingly prescribed for pharmacological GER treatment, even if pH-MII monitoring is rarely performed to confirm GER diagnosis [2]. These drugs have been associated with significant adverse effects, including an increased incidence of necrotizing enterocolitis and invasive infections [3, 4]. Hence, following appropriate diagnostic investigations, a stepwise therapeutical approach which firstly undertakes conservative strategies is the most advisable choice to avoid potentially harmful pharmacological overtreatments in the preterm population [5, 6].

Before 34 weeks’ corrected age, preterm infants are physiologically unable to coordinate sucking, swallowing, and breathing processes [7]; hence, intragastric tube feeding is often needed to ensure adequate enteral intakes. Continuous feeding and boluses are the enteral tube feeding techniques most used in neonatal care. Due to their different influence on gastric filling and emptying and to the prolonged permanence of the feeding tube through the lower esophageal sphincter (LES) required for continuous feeding, a potential impact of continuous and bolus feeding techniques on GER features has been hypothesized; available literature in preterm neonates, however, is lacking [8]. Hence, this study aimed to evaluate the effect of different techniques of enteral tube feeding on GER frequency and features in symptomatic preterm infants undergoing combined pH and multiple intraluminal impedance (pH-MII) for GER assessment.

## Materials and methods

Infants < 34 weeks’ gestation admitted to the IV level care NICUs of IRCCS AOU Bologna and ASST FBF-Sacco-Buzzi, Milan, Italy, were eligible for this observational prospective cross-over study if, due to GER symptoms (i.e., post-prandial regurgitations and/or desaturations, hiccup, fussing, back arching), a diagnostic 24-h pH-MII monitoring was required according to standard clinical practice and if fulfilling the following criteria at the time of pH-MII monitoring: enteral intakes > 100 ml/kg/day, need for tube feeding, weight > 1100 g, no need for invasive respiratory support or nasal CPAP.

The study was conducted in conformity with the Helsinki Declaration principles and approved by the local Ethics Committees (Ethics Committee of St. Orsola-Malpighi Hospital, Bologna, Italy, protocol ID: 119/2015/U/Oss; Comitato Etico Aziendale Milano Area A, Milan, Italy, ID 2016/ST/218). Written, informed consent was obtained from the infants’ parents or legal guardians.

The 24-h pH-MII monitoring (Sandhill Scientific Inc, Highland Ranch, Colorado) was performed as previously described [9] using a pH-MII probe (Comfortec pH-MII Sandhill Scientific Inc) with 7 impedance rings and 1 antimony electrode for pH detection, placed within the distal impedance dipole. The distance between the impedance rings was 1.5 cm, except for the distal dipole, which was 2 cm. The pH-MII probe was inserted trans-nasally; the probe position was calculated according to Strobel’s formula (esophageal length = 5 + 0.252 × height) and radiographically confirmed.

GER episodes were detected separately by pH monitoring and MII. GER episodes detected only by pH monitoring were defined as pH-GERs; reflux index (RIpH) identified the total percent time of esophageal exposure to pH < 4. GER episodes detected by MII (MII-GERs) were defined by a sequential impedance drop to less than 50% of baseline, starting distally and propagating backwards. According to the esophageal pH, MII-GERs were classified into acid (pH < 4, aMII-GER) or non-acid (pH ≥ 4, NaMII-GER). Bolus exposure index (BEI) identified the total percent time of esophageal exposure to MII-GERs and was further classified into acid and non-acid MII-GER-BEI. The maximum distance from LES reached by GER episodes into the esophageal lumen defined their migration height, according to which MII-GERs were classified as proximal (detected by the 1st, 2nd–3rd electrical dipoles) or distal (detected by the 4th and 5th electrical dipoles). Based on their physical features, MII-GERs were further defined as gaseous, liquid, and mixed.

**Fig. 1:**
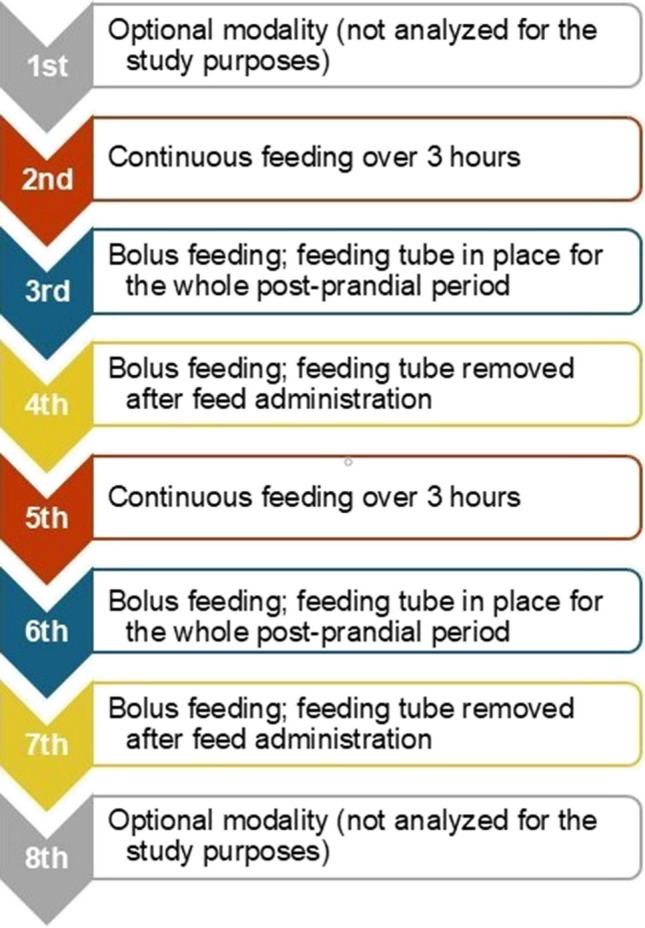
Sequence of meal administration during the 24-h pH-MII monitoring. Arrows indicate the order of study meals, while boxes highlight the meal modality

During the pH-MII monitoring, each enrolled infant received eight meals, administered with a 5-French orogastric feeding tube as they had not yet achieved full oral feeding. All enrolled infants were fed according to the sequence shown in Fig. 1 and followed the same meal sequence which was repeated twice: continuous tube feeding, bolus feeding followed by tube feeding permanence, bolus feeding followed by tube feeding removal. Bolus feeds were administered by gravity or through an infusion pump for a total duration of about 10 min; continuous feeds were administered over the course of 3 h. In order to minimize the effects of postures on the pH-MII evaluation, the enrolled infants were kept in supine position during and after the study meals. Moreover, each infant received the same type of milk (fortified maternal/donor human milk or formula) for the study feeds.

The BioVIEW Analysis software program (Sandhill Scientific Inc, v.5.7.1.0) was used to analyze pH-MII data. A direct visual evaluation of the pH-MII recording and of the layout of each GER episode was further performed by investigators blinded to the sequence of meal administration. A washout period was implemented between signal acquisition and meal ingestion, as the entire meal period following Ph-MII probe assembly and preceding Ph-MII probe disassembly were recorded but excluded from analysis (the first and eighth meals of the monitoring period).

### Statistical analysis

The required sample size (a minimum of 28 infants) was calculated at a one-tailed significance level of 5% and with a power of 80% to detect a 30% reduction in the number of non-acid GERs. Numerical variables were summarized as mean ± SD or median (interquartile range [IQR]) as appropriate; categorical variables were summarized as frequencies and percentages. The impact of the three feeding modalities on each pH-MII GER index was assessed using a multiple repeated-measures linear mixed model, which took into account the repeated measures obtained within each individual and performed pairwise comparisons between each feeding modality. Data analysis was performed using Stata software v.17 (StataCorp. 2017. Stata Statistical Software:Release 15. College Station, TX: StataCorp LP). Significance level was set at *P* < 0.05.

## Results

Between January 2016 and February 2023, thirty-one infants were enrolled; of these, one did not complete the pH-MII monitoring due technical issues; therefore, a total of 30 infants (IRCCS AOU Bologna, *n* = 20; ASST FBF-Sacco-Buzzi, *n* = 10) were included in the study analysis. Clinical characteristics of the study population are provided in Table 1.

**Table 1:** Clinical characteristics of the study population

Characteristics of the study population (*n* = 30)	
Gestational age (weeks), median (IQR)	29 (26.9–31)
Birth weight (g), median (IQR)	1291 (876–1573)
Intrauterine growth restriction, *n* (%)	3 (10)
Small for gestational age, *n* (%)	2 (7)
Sex (males), *n* (%)	15 (50)
Postmenstrual age at pH-MII monitoring (weeks), median (IQR)	34.6 (33.1–35.8)
Weight at pH-MII monitoring (g), median (IQR)	1685 (1514–2045)
Type of feeding at pH-MII monitoring, *n* (%)	
Exclusive, fortified human milk	6 (20)
Exclusive formula feeding	4 (13)
Mixed feeding	20 (67)
Volume of milk per meal (ml), median (IQR)	33 (30–40)
GER symptoms, *n* (%)	
Frequent regurgitations^a^	29 (97)
Recurrent postprandial desaturations^b^	18 (60)

*GER*, gastroesophageal reflux; *IQR*, interquartile range; *pH-MII*, pH-impedance monitoring

^a^ ≥ 3 regurgitations over a 12-h period

^b†^ ≥ 4 desaturations within 90 min after feeds over a 12-h period

Clinical indication to perform pH-MII monitoring were frequent regurgitations in 12/30 infants, recurrent post-prandial desaturations in 1/30, and both in 17/30. The pH-MII monitoring was well-tolerated by all the study infants. At the time of examination, none of them was receiving anti-reflux treatments and/or prokinetic drugs, while caffeine treatment was ongoing in 24/30 infants. None of the study infants received thickened human milk, anti-regurgitation or hydrolyzed formulas during the monitoring period.

GER features detected during continuous feeding and after the two bolus feeding modalities and the results of between-groups comparisons are detailed in Table 2.

**Table 2:** Gastroesophageal reflux (GER) features detected by pH-impedance (pH-MII) monitoring during/after each feeding modality; values are reported as median (IQR). *P*-values indicate the results of the multivariate linear mixed-effect models investigating the impact of feeding modalities on each GER index; significant pairwise comparisons are presented in bold

pH-MII parameters	Continuous feeding	Bolus feeding, FT in place	Bolus feeding, FT removed	*P*-value
*Number (n/24 h)*				
Total GERs Liquid GERs Gaseous GERs Mixed GERs pH-GERs aMII GERs NaMII-GERs	3 (0–6)*° 2 (0–6)*° 0 (0–0) 0 (0–0)*^§^ 0 (0–0)*° 0 (0–0)*° 2 (0–4)*°	11 (6–22)* 11 (4–21)*° 0 (0–0) 0 (0–2)* 2 (0–7)* 0 (0–2)* 8 (2–12)*	13 (8–21)° 13 (7–19)° 0 (0–0) 0 (0–2)^§^ 2 (0–9)° 1 (0–4)° 7 (3–12)°	** < 0.001** ** < 0.001** 0.361 **0.025** ** < 0.001** ** < 0.001** **0.009**
*Exposure indexes (%)*				
RipH aMII-GER-BEI NaMII-GER-BEI	0 (0–0)^†§^ 0 (0–0)^†^° 0.1 (0–0.4)*°	0.4 (0–2.3)^†^ 0 (0–0.2)^†^ 0.6 (0.1–1.6)*	0.4 (0–3)^§^ 0.04 (0–0.3)° 0.6 (0.3–1.1)°	**0.036** **0.009** **0.007**
*Mean duration (sec)*				
pH-GER aMII-GER NaMII-GER	87 (12–191) 15 (13–21) 16 (11–21)	54 (19–153) 17 (12–27) 18 (13–28)	59 (24–99) 20 (14–31) 20 (15–32)	0.101 0.301 0.239
*Migration height (n/24 h)*				
Proximal GER, total Proximal GER, aMII Proximal GER, NaMII Distal GER, total Distal GER, aMII Distal GER, NaMII	0 (0–2)*° 0 (0–0)^†^° 0 (0–1)*° 1 (0–4)*° 0 (0–0)*° 0 (0–2)*°	3 (1–7)* 0 (0–1)^†^ 2 (1–6)* 7 (3–15)* 3 (0–8)* 3 (1–6)*	4 (1–8)° 0 (0–1)° 3 (1–5)° 9 (3–15)° 3 (0–11)° 3 (1–6)°	** < 0.001** ** < 0.001** **0.008** ** < 0.001** ** < 0.001** **0.005**

†,§*P* < 0.05; *,°*P* < 0.01

*aMII*, acid MII; *FT*, feeding tube; *NaMII*, non-acid MII; *RipH*, reflux index

A significantly lower number of total, liquid, mixed GER episodes was observed during continuous feeding compared to bolus feeds, either followed by tube feeding permanence or removal, while no difference was observed between the two bolus feeding techniques. Compared with bolus feeds, continuous feeding was also associated with a lower number of pH-GER, aMII-GER, and NaMII-GER episodes; similarly, RiPH, aMII-GER-BEI, and NaMII-GER-BEI were significantly lower during continuous feeding, whereas no difference was observed in the mean duration of each pH, aMII, and NaMII-GER episode. The frequency of pH-MII, aMII, and NaMII-GERs, their mean duration, and the related exposure indexes did not differ between bolus feeds followed by tube feeding permanence or removal. Both bolus feeding modalities were associated with a slightly but significantly higher number of total, aMII, and NaMII proximal GERs compared to continuous feeding. The number of total, aMII, and NaMII distal GER episodes was also increased following bolus feeds with and without feeding tube removal, while no difference in the frequency of proximal and distal GER episodes was observed between the two methods of bolus feeding.

## Discussion

This study compared multiple pH-GER parameters in relation to different tube feeding methods in symptomatic preterm infants undergoing pH-MII monitoring. Our findings suggest that continuous feeding may decrease the frequency and the esophageal height of acid and non-acid GER episodes in tube-fed preterm neonates compared to bolus feeding. However, the permanence of the intragastric feeding tube after bolus administration did not significantly impact pH-MII GER characteristics.

It is important to note that the overall incidence of reflux episodes and acid exposure was low in our cohort, suggesting that many infants may not have had pathological GER. This limitation may affect the generalizability of our results to infants with more severe reflux or specific comorbidities.

Due to the important adverse effects correlated with the administration of anti-reflux drugs in preterm infants, non-pharmacological strategies, such as postural and dietary interventions, are considered the first-line approach to manage GER in this population. Understanding the impact of routine care practices (e.g., non-nutritive sucking or different feeding techniques) [10–13] on GER symptoms and characteristics is crucial to prevent GER exacerbation in symptomatic neonates. Given the pathogenic role of delayed gastric emptying and transient LES relaxations on neonatal GER [14–16], different feeding duration, as well as the permanence of the intragastric tube through the LES, may influence GER patterns.

Due to the immaturity of their oral feeding competences, preterm infants often require intragastric tube feeding in order to achieve adequate enteral intakes. Two common methods are bolus and continuous feeding. Bolus feeding involves the administration of the meal by gravity or through an infusion pump for a total duration of about 10 min, after which the feeding tube can be removed. While this method mimics physiological feeding patterns by stimulating cyclical surges gastrointestinal hormones [17] and increasing splanchnic oxygenation during the post-prandial period [18], the short time elapsing between tube insertion for bolus administration and its subsequent removal might trigger a vagal response, characterized by a transient bradycardia which may lead to a subsequent temporary reduction of cardiac output [19]. Moreover, the rapid gastric distension that follows the administration of a large bolus feed could increase the pressure throughout the LES, thus contributing to the refluxate of gastric contents into the esophageal lumen.

On the other hand, continuous feeding provides a steady, slower administration of enteral feeds over a longer period (e.g., 2 or 3 h). Although this technique has been associated with a greater delay in reaching full enteral feeds when compared with intermittent boluses [20], it has also been reported to improve the absorption of nutrients and to hasten gastric emptying in preterm infants [21, 22]. Data from healthy adults showed that bolus nasogastric feeding led to faster gastric emptying compared to continuous feeding [23]. However, studies on duodenal motor responses to feeding of different nutrient content and rate of feeding in preterm infants showed that only full-strength formula given as slow infusion increased duodenal motor activity and triggered adult-like duodenal motor responses to feeding [24]. Hence, continuous feeding may be preferable in preterm infants in case of delayed gastric motility, persistent gastric residuals and abdominal distension; moreover, by causing less gastric distension, it could be associated with lower LES pressure. However, this technique requires the permanence of the feeding tube through the LES for the whole feeding period, and this may have a theoretical impact on GER occurrence.

To the best of our knowledge, this is the first prospective study comparing GER features, assessed using pH-MII monitoring, between 3-h continuous feeding and bolus feeds in preterm neonates, and investigating the possible impact of tube feeding removal following bolus administration. A similar study, performed on healthy adult volunteers without any gastrointestinal motility disorders, reported no difference in gastric emptying and in GER frequency and duration [25]; however, the lack of GER symptoms in the study population as well as the greater anatomical competence of LES in the adult population makes these results not comparable to the present ones.

According to our results, continuous feeding was associated with a significant reduction of both acid and non-acid GER frequency and with a lower esophageal GER migration when compared to bolus feeds, either followed by the removal of tube feeding or not. This may be due to the slow infusion rate that characterizes this feeding technique, which prevents a rapid gastric distension, and the subsequent pressure increase on the LES, which could trigger not only GER onset but can also favor proximal migration of the refluxate. A similar decrease in the number of total and non-acid GER events at increasing feeding durations has been previously described in dysphagic neonates assessed by pH-MII [26]. Favara et al. previously compared GER frequency and acid exposure between bolus feeds and feeds administered over ≥ 60 min, observing a significant decrease in non-acidic GER frequency, but not in esophageal acid exposure nor in GER symptoms, with a longer feeding time [12]. However, this was a retrospective study, and the median feeding duration in the prolonged feeding group was not reported; moreover, whether the feeding tube was removed or left in place following each meal administration was not clarified. Ibrahim et al. compared several gastrointestinal outcomes, including GER, between 2 vs. 3-h continuous feeding using a randomized-controlled study design; in their trial, however, GER was defined clinically as unexplained apnea or bradycardia requiring anti-reflux treatment, and no instrumental assessment was performed to assess GER features in relation to the two feeding periods [11]. Other prospective, randomized clinical trials compared bolus vs. continuous feeding in preterm infants, but GER was not included among the assessed outcomes [20, 27–29].

As shown in earliest adult evidence in which this technique was used to heal peptic ulcers and to relieve the related pain [30, 31], continuous intragastric milk drip can effectively buffer acid gastric secretion. Consistently, in the present study, acid refluxes and the related acid exposure indexes were almost eliminated during continuous feeding. Due to the buffering effects of milk feeds, in the preterm population, non-acid GER tends to prevail [32], and this may have contributed to the overall low number of acid GERs observed in the present study. In tube-fed infants with symptoms related to proximal GER migration or with a predominant acid GER component, our findings may support the beneficial role of continuous feeding, which could help to limit the use of antiacid drugs in few, selective cases.

In the present study, removing or maintaining the feeding tube in place following bolus administration did not influence pH-MII GER frequency nor the esophageal GER height; moreover, the improved GER indexes observed during continuous feeding suggest that the feeding tube permanence across LES for the whole feeding period had no harmful effects. Murthy et al. compared GER frequency between infants with or without a 5-French nasogastric feeding tube, showing significantly increased GER events in the latter group following both bolus and more prolonged feeds [13]; this, however, was a retrospective study without a cross-over design, and the two study groups significantly differed in terms not only of gestational age and birth weight but also of corrected age, weight, and fluid intakes at the time of the study, thus underlying a potential bias. We have previously evaluated the effect of bolus and continuous feeding on the incidence of cardio-respiratory events, which could represent clinical atypical manifestations of GER [33], observing a slight but significant increase of apneic episodes during continuous feeding; however, the effect of tube feeding removal or permanence after bolus administration was not investigated [34]. Although it could not be established from the current data, the present findings suggest that the increased frequency of cardiorespiratory events previously described during continuous feeding may not represent a clinical GER manifestation but could rather reflect a possible side effect of the feeding tube placement and permanence, especially if inserted via the nasogastric route [35].

Limitations of this study include the small sample size and the fixed meal sequence for all enrolled infants, which could introduce an “order effect.” However, using a cross-over design where each infant served as their own control mitigated the effect of possible clincal confounding factors.

The study design did not allow to assess a possible correlation between the 3 feeding techniques, the documented GER episodes, and the occurrence of such cardiorespiratory events as apneas or bradycardias. Additionally, the present study did not assess the impact of different feeding techniques on GER symptoms. Future research is needed to determine if these feeding methods influence symptom presentation. It is crucial to recognize that pH-MII measurements may not always correlate directly with clinical symptoms and outcomes.

According to our data, continuous feeding and bolus feeding followed by tube feeding permanence or removal have a different impact on pH-MII GER features in tube-fed preterm infants. When compared to bolus feeding techniques, continuous feeding was associated with a significant decrease in total, acid, and non-acid GER frequency and in a lower esophageal migration of the refluxate. Moreover, the permanence of the feeding tube across LES during or after the feeding period did not seem to worsen GER occurrence. Adopting continuous feeding in tube-fed preterm infants with GER can aid to reduce pH-MII GER episodes and, along with other conservative strategies, may contribute to limit the use of potentially harmful antiacid medications in this population; nevertheless, further larger studies are needed to validate these data and to evaluate the effect of different tube feeding techniques on GER-related symptoms.

## Data Availability

The datasets generated during and/or analysed during the current study are available from the corresponding author on reasonable request.

## Abbreviations

LES: Lower esophageal sphincter
GER: Gastroesophageal reflux
pH-MII: Combined pH and multiple intraluminal impedance monitoring
NICU: Neonatal intensive care unit
CPAP: Continuous positive airway pressure
pH-GERs: Gastroesophageal reflux episodes detected only by pH monitoring
RIpH: Reflux index
MII-GERs: Gastroesophageal reflux episodes detected by MII
aMII-GER: Acid MII-GER
NaMII-GER: Non-acid MII-GER
BEI: Bolus exposure index
